# Research status and progress of radiomics in bone and soft tissue tumors: A review

**DOI:** 10.1097/MD.0000000000036198

**Published:** 2023-11-24

**Authors:** Xiaohan Zhang, Jie Peng, Guanghai Ji, Tian Li, Bo Li, Hao Xiong

**Affiliations:** 1 a Department of Radiology, The First Affiliated Hospital of Yangtze University, Jingzhou, China.

**Keywords:** radiomics, bone and soft tissue tumors, computed tomography, magnetic resonance imaging

## Abstract

Bone and soft tissue tumors are diverse, accompanying by complex histological components and significantly divergent biological behaviors. It is a challenge to address the demand for qualitative imaging as traditional imaging is restricted to the detection of anatomical structures and aberrant signals. With the improvement of digitalization in hospitals and medical centers, the introduction of electronic medical records and easier access to large amounts of information coupled with the improved computational power, traditional medicine has evolved into the combination of human brain, minimal data, and artificial intelligence. Scholars are committed to mining deeper levels of imaging data, and radiomics is worthy of promotion. Radiomics extracts subvisual quantitative features, analyzes them based on medical images, and quantifies tumor heterogeneity by outlining the region of interest and modeling. Two observers separately examined PubMed, Web of Science and CNKI to find existing studies, case reports, and clinical guidelines about research status and progress of radiomics in bone and soft tissue tumors from January 2010 to February 2023. When evaluating the literature, factors such as patient age, medical history, and severity of the condition will be considered. This narrative review summarizes the application and progress of radiomics in bone and soft tissue tumors.

## 1. Introduction

Bone and soft tissue tumors are less common than other types of tumors in other systems, with more complex histological composition and biological behaviors, and overlap among different types of tumors in imaging manifestations, which hinder radiologists to assess imaging features with naked eyes and make diagnoses relying on clinical data and personal experience. However, radiomics can extract subvisual quantitative features from medical images and then analyze them, which has a distinct advantage that is tough to achieve with traditional imaging methods, so as to guide clinical decision-making and precision medicine.

In 2012, Lambin P, a Dutch scientist, first put forward the concept of radiomics,^[1]^ and defined it as the high-throughput extraction of a large number of imaging features from region of interest (ROI) of images. Kumar et al^[2]^ further refined the concept of radiomics, that is, using high-throughput methods to extract and analyze quantitative imaging features from computed tomography (CT), magnetic resonance (MR), and positron emission tomography (PET) images, so as to diagnose diseases and evaluate the prognosis of diseases, thus began the research of radiomics.

**Figure 1.**
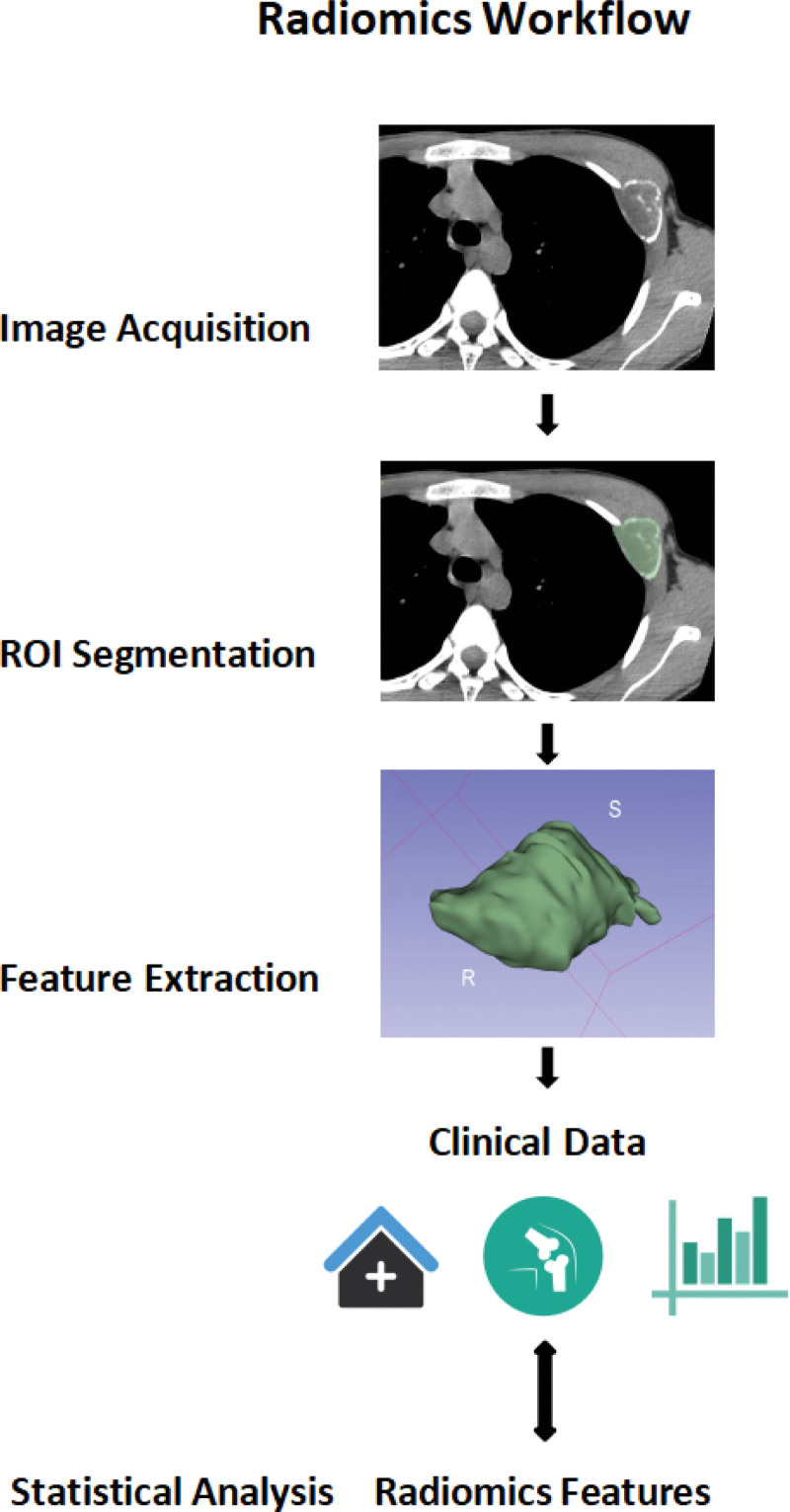
The radiomics workflow.

The workflow of radiomics was summarized as follows: Obtaining high-quality and standardized images. Defining ROI from conventional medical images, that is, the segmentation of lesions. The segmentation includes manual, semi-automatic, and fully automatic methods. ITK-SNAP, 3D Slicer, and ImageJ are 3 popular software. Extracting various imaging features (intensity, morphology, texture, and wavelet) from software and realizing dimensionality reduction.^[3]^ Establishing a model using machine learning. The final objective is to provide accurate risk stratification by a predictive model, and to assess how these valuable features can be widely regarded as predictors. The radiomics workflow is displayed in Figure 1.

Radiomics is not more intelligent than people, but the information extracted by naked eyes is always shape-based and structure-based. The features extracted from radiomics are intensity, texture, wavelet, maximum value, and standard deviation, which cannot be seen by human eyes and are difficult to process by human brain. That is precisely what computers excel at. Therefore, in the feature selection process, the features selected by computers and the features recognized by naked eyes form a complementary relationship. If we can use computers to extract high-dimensional features, and then integrate empirical features, text information, genetic information, etc, it must be “1 + N>N.” We can accomplish human-computer interaction and realize precision medicine. Another extremely significant point is that these high-dimensional features contain microscopic information. In the past, naked eyes were unable to extract it. Thanks to advances in computer technology, now it can be retrieved for systematic analysis, improving the accuracy of our predictions.

Feature extraction is a key step in radiomics analysis, which directly affects the results of subsequent radiomics analysis. Therefore, feature extraction algorithms should extract features that can effectively reflect tumor information or are related to clinical labels and minimize redundant features.

Machine learning from big data is necessary for the development of the radiomics model in the field of bone and soft tissue tumors. Using the program code, machine learning enables computers to simulate or realize the learning behavior of humans in order to gain new knowledge or skills. Common machine learning methods include: unsupervised machine learning, semi-supervised machine learning, supervised machine learning and reinforcement learning. Popular machine learning algorithms in medicine include linear regression, logistic regression, decision trees and random forests.^[4]^

With the rise of radiomics, a series of domestic and international studies have been conducted, which are related to the application of radiomics in the nerve, breast, chest, abdomen and pelvic. However, relatively few studies have concentrated on the application of radiomics in the musculoskeletal system. The present study aimed to explore the pipeline, clinical applicability and current status, and highlight the challenges of radiomics in bone and soft tissue tumors.

## 2. Application of radiomics in bone and soft tissue tumors

As far as bone and soft tissue tumors are concerned, information obtained from visual observation is limited, and radiomics is advantageous to assist radiologists to diagnose a variety of diseases. It considers images as quantitative data for data extraction, assists radiologists and clinicians to better understand the mechanisms of diseases, and improves medical care standards, including evaluating the efficacy of neoadjuvant chemotherapy before surgery, avoiding overtreatment, predicting local recurrence and distant metastasis after surgery, etc.^[5]^

### 2.1. Differential diagnosis of bone tumors

The role of radiomics in differentiating bone tumors with similar manifestations in CT images has noticeably attracted clinicians’ attention in recent years. A number of studies have shed light on the differential diagnosis of chordoma and giant cell tumor of bone using CT images. Yin et al^[6]^ established a radiomics model based on 3-dimensional (3D)-CT and CT enterography (CTE) to identify the most effective machine learning method for differentiating between sacral chordoma and sacral giant cell tumors. The most effective approach was determined to be the least absolute shrinkage and selection operator (LASSO) classifier plus generalized linear model. A study used CT-based radiomic features to distinguish focal and diffuse multiple myeloma (MM). Regarding assessment of imaging features, it was reported that 15% of features in diffuse and focal patterns differed. The present study found that a radiomics approach outperformed radiologists’ discrimination accuracy when it was applied to discriminate focal from diffuse patterns.^[7]^ Nie et al^[8]^ used a multivariate logistic regression model to combine Rad score with independent clinical risk factors, established radiomics nomogram, and realized preoperative differentiation between chordotoma and giant cell tumor (GCT) in axial bone.

To date, MRI-based radiomics has been preliminarily applied in the differential diagnosis of bone tumors. For similar MR images manifesting skull base chordoma (SBC) and chondrosarcoma, discriminating them using preoperative images is a diagnostic challenge.^[9]^ Some scholars gathered MR images (TIWI, T2WI, CET1) of 154 patients suffering from SBC and 56 patients with chondrosarcoma and extracted multi-parametric MRI (mp-MRI) radiomics features. The single-sequence MRI radiomics model exhibited a worse performance than the mp-MRI radiomics model, where the values of area under the receiver operating characteristic curve (AUC) reached 0.97 and 0.87, respectively. The research has shown that the radiomics model based on mp-MRI before surgery could accurately and reliably identify chordoma with chondrosarcoma of the skull base, thereby assisting clinicians to distinguish diverse types of tumors preoperatively and develop an individualized treatment plan.^[10]^ Moreover, a multiparametric MRI-based radiomics signature can accurately distinguish between osteosarcoma and Ewing sarcoma. For a radiomics model based on T2-FS and CET1, the AUC values were 0.881 and 0.765, respectively.^[11]^

In recent years, radiomics based on Positron Emission Tomography/ Computed Tomography (PET/CT) and Single-Photon Emission Computed Tomography/ Computed Tomography (SPECT/CT) has also advanced in the differential diagnosis of bone tumors. Nowadays, several studies have demonstrated the satisfactory diagnostic performance of radiomic models based on 18F-fluorodeoxyglucose (18F-FDG) PET/CT images for the classification of MM and bone metastases. The study conducted by Mannam et al^[12]^ aims to evaluate the diagnostic ability of models based on first- and second-order texture features of PET and CT images in differentiating bone metastases from MM. Jin et al^[13]^ also discussed the diagnostic performance of radiomic model constructed based on PET/CT images for multiple myeloma and bone metastasis. Moreover, compared with SUVmax, the conventional PET parameter, the radiomic model showed significant improvement in diagnostic performance. Also, there are a few studies based on SPECT/CT-based radiomics in relation to bone diseases. Jin^[14]^ discovered that radiomics derived from SPECT/CT images are capable of efficiently differentiating between benign bone lesions and bone metastases. This technique could be a new noninvasive alternative to assist in avoiding needless diagnostic delays.

With the development of artificial intelligence, there have been advances in the application of CT-based radiomics for differentiating bone metastases from non-neoplastic lesions. A quantitative approach was also proposed to evaluate the diagnostic efficacy of differentiating bone islands from osteoblastic bone metastases by Hong et al,^[15]^ who selected 10 radiomics features to establish a random forest (RF) model and carried out 10-fold cross-validation. Studies have revealed that, compared with inexperienced radiologists, the CT-based RF model could effectively distinguish bone islands from osteoblastic bone metastases, and the diagnostic performance was equivalent to that of experienced radiologists. Previous research indicated that the weighted k-nearest neighbors approach showed the most optimal performance (the highest accuracy: 73.5%; AUC: 76%). CT textural analysis (CTTA) and machine learning methods were utilized to differentiate metastatic lesions from completely responded sclerotic bone lesions in prostate cancer (PCa) via PET.^[16]^

Due to the low incidence and diversity of cartilage tumors, the specificity of their diagnostic imaging is low. Several studies have employed radiomics to obtain more valuable information. Both radiologists and pathologists attempted to distinguish low-grade chondrosarcoma from chondroma.^[17]^ Radiomics based on mp-MRI (T1-weighted (T1W), contrast-enhanced T1W (CET1W), T2-weighted (T2W) and short tau inversion recovery (STIR)) has yielded promising results. This finding clearly indicated that kurtosis in the contrast-enhanced T1W has the maximum discriminative ability, as well as high sensitivity, specificity, and accuracy.^[18]^ A study included patients with cartilaginous bone tumors, and bidimensional segmentation of T1W MR images was carried out. According to the study, ACT and CS2 of long bones could be classified with high accuracy using machine learning methods based on MRI.^[19]^

It is noteworthy that the value of radiomics in the differentiation of bone muscle tumors is not limited to dichotomous classification. It is equally applicable to the differentiation among 3 or more types of tumors. Yin et al^[20]^ analyzed T2WFS and CET1W images of 120 patients with sacral tumors (sacral chordoma, sacral giant cell tumor, and sacral metastatic tumor) that were pathologically confirmed, and established a RF-based radiomics model after filtering out features using LASSO regression algorithm. Features extracted from T2WFS and CET1W images performed better compared with those retrieved from either T2WFS or CET1W images. Compared with features extracted from CET1W images, those retrieved from T2WFS images reached higher AUC values. The AUC of the model established by combining T2WFS and CET1W features could reach 0.773. A recent study has shown that a machine learning model using radiomics features and demographic data could significantly distinguish benign from malignant bone tumors via X-ray images. Artificial neural network (ANN) yielded the greatest outcomes, outperforming radiology residents in terms of accuracy.^[21]^ Chianca et al^[22]^ tested the diagnostic performance of machine learning in the differential diagnosis of spinal bone lesions using 2 different software (HeterogeneityCAD module in 3D Slicer (hCAD) and PyRadiomics) to acquire radiomics data. Lesions were labeled as benign or malignant (two-label classification), and benign, primary malignant or metastatic (three-label classification). The hCAD and PyRadiomics extracted 90 and 1548 features, respectively. For the 2-label classification, hCAD data achieved accuracy of 94% and 86% in the test datasets, respectively. For the 3-label classification, PyRadiomics data achieved accuracy of 80% and 69% in the test datasets, respectively. Collectively, these results indicated that although qualitative images still play a central role in the differential diagnosis of tumors, machine learning models based on radiomics are currently promising tools.

The main characteristics of the studies discussed in Sections 2.1 are detailed in Table 1.

**Table 1:** Performances of radiomics models in differential diagnosis of bone tumors and soft tissue tumors.

Authors	Yr	Study design	Type of tumor	Technique	No. of patients (training + testing)	Performances
Yin et al^[6]^	2019	Retrospective	Chordoma & Giant cell tumor	CT	95	AUC = 0.79
Tagliafico et al^[7]^	2021	Retrospective	Multiple myeloma	CT	70	AUC = 0.79
Nie et al^[8]^	2023	Retrospective	Chordoma & Giant cell tumor	CT	111	AUC = 0.980
Li et al^[10]^	2019	Retrospective	Chordoma & Chondrosarcoma	MR	210	AUC = 0.87
Dai et al^[11]^	2020	Retrospective	Osteosarcoma & Ewing sarcoma	MR	66	AUC = 0.77–0.88
Mannam et al^[12]^	2023	Retrospective	Skeletal metastases & Multiple myeloma	PET/CT	40	AUC = 0.945 AUC = 0.9538
Jin et al^[13]^	2022	Retrospective	Spine multiple myeloma & Bone metastasis	PET/CT	131	AUC = 0.973
Jin et al^[14]^	2021	Retrospective	Bone metastases & Benign bone lesions	SPECT/CT	132	AUC = 0.951
Hong et al^[15]^	2021	Retrospective	Osteoblastic metastases	CT	177	AUC = 0.96
Acar et al^[16]^	2019	Retrospective	Metastatic and completely responded sclerotic bone lesion	PET/CT	75	AUC = 0.76
Lisson et al^[18]^	2018	Retrospective	Enchondroma & Chondrosarcoma	MR	22	AUC = 0.851, 0.822 AUC = 0.876, 0.826
Gitto et al^[19]^	2022	Retrospective	Atypical cartilaginous tumor & Chondrosarcoma	MR	158	AUC = 0.94
Yin et al^[20]^	2018	Retrospective	Chordoma & Giant cell tumor & Metastatic tumor	MR	120	AUC = 0.773
Schacky et al^[21]^	2022	Retrospective	Bone tumors	X-ray	880	AUC = 0.79 AUC = 0.90
Chianca et al^[22]^	2021	Retrospective	Bone tumors	MR	146	2-label: AUC = 0.94, 0.86 3-label: AUC = 0.80, 0.69
Juntu et al^[23]^	2010	Retrospective	STTs	MR	135	AUC = 0.91
Kim et al^[24]^	2017	Retrospective	Myxoid-containing STTs	MR	40	AUC = 0.923
Malinauskaite et al^[25]^	2020	Retrospective	Lipoma & Liposarcoma	MR	38	AUC = 0.926
Yue et al^[26]^	2022	Retrospective	STTs	MR	139	AUC = 0.923
Xu et al^[27]^	2014	Retrospective	Bone and soft tissue lesions	PET/CT	103	Sensitivity = 86.44 % Specificity = 77.27 % Accuracy = 82.52 %

STTs = soft tissue tumors.

### 2.2. Differential diagnosis of soft tissue tumors (STTs)

There are several types of STTs, as well as some challenges in the clinical, imaging, and pathological differentiation. The main treatment is surgery, supplemented with radiotherapy, chemotherapy, and targeted therapy when necessary. Preoperative imaging examination to determine the grade of STTs is an important part of the treatment plan.

Certain soft tissue sarcomas (STSs) have comparable appearances in medical images between benignancy and malignancy. The phenomenon frequently leads to delayed diagnosis and initial management.^[28]^ If malignant tumors can be diagnosed efficiently in time, patients’ survival rate can be elevated substantially.^[28,29]^ To date, a series of MRI-based radiomics studies have concentrated on the identification of STTs. A large-scale, single-center study conducted by Juntu et al considered radiomics-based texture heterogeneity and used 3 traditional machine learning classifiers. They found that support vector machine (SVM) classifiers could significantly distinguish benign from malignant STTs, with AUC values of 0.92 and 0.85, respectively, for SVM and radiologist diagnosing STTs.^[23]^ Nevertheless, the study only included a portion of tumor regions, failing to thoroughly replicate the tumor characteristics. The other researchers progressively improved the ROI selection process. For instance, Kim et al^[24]^ segmented MR images of patients suffering from myxoid-containing STTs layer by layer to acquire first- and second-order features of ROI. They found a more homogeneous spatial pattern on apparent diffusion coefficient (ADC) maps in malignant myxoid-containing STTs than those in the benign myxoid-containing STTs, confirming the significance of radiomics based on functional MRI in the qualitative diagnosis of STTs.^[30]^ The treatment, follow-up and prognosis of patients with these 2 diseases are markedly different, and distinguishing lipoma from liposarcoma only by conventional MRI examination is a diagnostic challenge. Given the robustness of T1WI sequences, Malinauskaite et al^[25]^ used them in their study. Using preoperative MRI, they assessed the capacity of radiomics and machine learning to distinguish soft tissue lipoma from liposarcoma. Apart from this, they compared the diagnostic accuracy of the machine learning model with that of 3 radiologists specialized in the musculoskeletal system. With an AUC of 0.926, they found that the machine learning classifier generated the best outcome. More recently, Yue et al^[26]^ showed the clinical potential of radiomics based on multi-parameter MRI in distinguishing benign and malignant soft tissue tumors, which can be considered as a noninvasive personalized therapy tool.

The field of radiomics has grown rapidly, and PET/CT-based radiomics is no exception.^[31]^ Xu et al^[27]^ analyzed the textural features of 18F-FDG PET/CT images of patients with benign and malignant lesions of bone and soft tissue. They developed 3 types of computer-aided diagnosis (CAD) methods: a CT-based textual analysis-only method, a PET-based textual analysis-only method, and a combined model (PET + CT). Their differential diagnostic capabilities were compared with the standardized uptake value (SUV) method. It was found that the combined model (PET + CT) exhibited an excellent diagnostic performance compared with the SUV method.

The main characteristics of the studies discussed in Sections 2.2 are detailed in Table 1.

### 2.3. Grading of bone and soft tissue tumors

Noninvasive quantification of tumor heterogeneity is an emerging, promising and challenging research field. Regarding tumor heterogeneity across the tumor volume, radiomics features may offer complementary information.^[32]^ Formulating treatment strategies critically depends on preoperative tumor grade prediction.

STSs are classified as low, medium or high grade, and the prognosis varies by grade. Moreover, determining the tumor grade is crucial in selecting therapeutic options, especially in distinguishing between low grade (G1) and high grade (G2 or G3).^[33]^ Macroscopic intratumoral heterogeneity can be judged by visual observation of hemorrhage, necrosis and calcifications within the lesion, whereas microscopic heterogeneity advocates the need for more refined diagnostic tools, and the application of radiomics to grade STS has been a hot topic in recent years. Some scholars extracted features from T1WI and fast spin echo images by establishing the radiomics model. They found that the machine learning based on radiomics could be used to distinguish low-level STS from high-level STS preoperatively.^[34]^ Another radiomics nomogram was then developed by fusing the radiomics signature with clinical staging. The AUCs of the 3 radiomics models based on T2FS, T1FSGd and a combined model in independent validation set were 0.78, 0.69, and 0.76, respectively. It has shown that the combination of radiomics-T2FS and the AJCC clinical staging system is capable of achieving the optimal grading performance, and it can be a potential tool to differentiate low-grade STS from high-grade STS.^[35]^ PET/MR has begun to be used in the diagnostic evaluation of adults and children with soft tissue sarcomas, as one of the most recent technological advancements that we are able to implement in our practice.^[36]^ With less radiation exposure than PET/CT, this method also gives metabolic data, in addition to permit the simultaneous acquisition of quantitative MR data.^[37]^ The first PET/MR-based radiomics for pediatric soft tissue sarcoma was presented by Giraudo et al^[38]^ This study showed that the radiomic features lmc1 and variance can distinguish between pediatric soft tissue sarcomas of different grades and histotypes. As for adults, Corino et al^[39]^ found comparable findings.

As seen for STS, the grade of cartilaginous tumor has a noticeable influence on clinical outcomes. The 10-year survival rate for low-grade/atypical chondrogenic tumors is 88%, while the 10-year overall survival (OS) rates for grade 2 and 3 chondrosarcomas dropp sharply to 62% and 26%, respectively.^[40]^ Furthermore, treatment regimens noticeably vary among different grades of cartilaginous tumors.^[41]^ Preoperative biopsies are subjected to sampling errors and a significant interobserver variability of the pathologist report, all of which can lead to inaccuracy in the downgrading of cartilaginous tumors, therefore, new methods are urgently required to evaluate their grading.^[42]^ Encouragingly, several MRI-based radiomics models have confirmed machine learning and textural analysis as new methods to differentiate various cartilage tumor grades. Attention was paid to the differential diagnosis of low-to-high grade cartilaginous bone tumors by Gitto et al They extracted 172 features from MR images. Emerging evidence indicated that 4 radiomics features exhibited an excellent diagnostic performance.^[43]^ Another similar study demonstrated how intratumoral heterogeneity on MRI can help discriminate different grades of cartilaginous bone tumors. Benign and malignant lesions could be distinguished with a diagnostic accuracy of 92.9%, and benign and low-grade lesions could be distinguished with a diagnostic accuracy of 91.2%, using predictor models that included morphological MRI characteristics and textural analysis features. A growing body of evidence demonstrated that, as opposed to morphological MRI analysis, textural analysis could improve diagnostic accuracy for differentiating benign from malignant lesions, as well as benign from low-grade cartilaginous lesions.^[44]^

The main characteristics of the studies discussed in Sections 2.3 are detailed in Table 2.

**Table 2:** Performances of radiomics models in grading of soft tissue tumors.

Authors	Yr	Study design	Type of tumor	Technique	No. of patients (training + testing)	Performances
Wang et al^[34]^	2020	Retrospective	STSs	MR	113	C-Index = 0.96
Peeken et al^[35]^	2019	Retrospective	STSs	MR	225	AUC = 0.78 AUC = 0.69 AUC = 0.76
Giraudo et al^[38]^	2022	Retrospective	STSs	PET/MR	18	Accuracy = 70.4%
Corino et al^[39]^	2018	Retrospective	STSs	MR	19	AUC = 0.85, 0.87
Gitto et al^[43]^	2020	Retrospective	Cartilaginous bone tumors	MR	58	C-index = 0.78
Fritz et al^[44]^	2018	Retrospective	Cartilaginous bone tumors	MR	116	Accuracy = 92.9%, 91.2%

STSs = soft tissue sarcomas.

### 2.4. Treatment response in bone and soft tissue tumors

Radiomics features can be used for predicting clinical endpoints, such as treatment response, which are able to detect early disease-related imageology variety.^[32]^

Predicting treatment response to osteosarcoma has long been a question of great interest in a wide range of fields. Traditional radiomics features extracted from single-phase medical images ignore changes in the course of treatment or follow-up, while delta-radiomics quantifies changes in radiomics features during or after treatment, thus, it is more appropriate for the evaluation of treatment response.^[45]^ Lin et al^[46]^ retrospectively analyzed CT images of patients with high-grade osteosarcoma who underwent neoadjuvant chemotherapy. After drawing ROI on CT images before and after chemotherapy, they established a radiomics model combining radiomics features with clinical factors. The delta-radiomics features showed a greater AUC than those based on a single CT sequence, which can be used to assess pathological response following preoperative chemotherapy and contribute to formulating appropriate chemotherapy regimens and treatment plans. Jeong et al acquired 18F-FDG PET images of osteosarcoma patients before and after neoadjuvant chemotherapy (NAC), and a retrospective study was then conducted to evaluate the association between 18F-FDG textural features and chemotherapy response, so as to determine consequent information.^[47]^ A previous study assessed the differentiation accuracy of mp-MRI and machine learning for evaluating tumor necrosis in osteosarcoma patients who underwent neoadjuvant chemotherapy. In addition, mp-MRI and machine learning proved an excellent differentiation performance. The mentioned study indicated the need for combination of mp-MRI and machine learning in order to differentiate tumor necrosis from tumor survival dramatically.^[48]^ In addition, a nomogram made up of clinical factors and an MRI-based radiomics score supports its use in predicting how well patients with osteosarcoma would respond to neoadjuvant chemotherapy (NAC).^[49]^ Nuclear medicine images have also been used to analyze the results of chemotherapy responses.^[50]^ Kim et al^[51]^ discovered that a prediction model combining radiogenomics approaches in conjunction with PET/CT imaging features and gene expression could reliably predict treatment response.

Radiomics is a noninvasive and more comprehensive approach to assess treatment response in patients with STS.^[52]^ NAC based on anthracyclines improves survival of patients with STS. At present, personalized treatment for patients with STS is dependent on pathological findings, and the emergence of radiomics has overcome this limitation. Radiomics based on both conventional MRI and functional MRI has been preliminarily reported in predicting treatment response. For instance, Crombé et al^[53]^ tested radiomics on MR images of 65 patients with STS. All patients received NAC after surgery and underwent MRI examinations at baseline and after 2 cycles of chemotherapy to outline the full layers of ROI from T2WI sequences. Combining RF classifier with T2WI imaging features exhibited the best performance, which has a potential application value in predicting response to neoadjuvant chemotherapy. Gao et al^[54]^ obtained radiomics features from longitudinal diffusion-weighted MRI (DWI) and developed a SVM model to predict pathologic treatment effects on patients with localized STS undergoing preoperative radiotherapy. SVM was found noticeably superior to logistic regression, and it was found that mean ADC, delta ADC and radiomics features alone were insufficient to predict response. Blackledge et al followed up STS patients for 2 to 4 weeks after radiotherapy using a supervised machine learning approach to monitor changes after radiotherapy. They discovered that when Naïve-Bayes was combined with Markov Random Field method was successful in visualizing and quantifying changes in independent STS subtypes after radiotherapy and in assessing heterogeneous responses.^[55]^ In contrast to size, density and perfusion, Tian et al^[56]^ evaluated the role of CTTA in evaluating the response of STS after treatment with neoadjuvant bevacizumab (BVZ) combining radiotherapy. This study also sheds new light on a unique biomarker for the treatment response of STS.

In recent years, there has also been a significant progress in the study of radiomics models to predict treatment response of other tumors. One study used radiomics approach to evaluate immune infiltration of tumors and proved that radiomics signature is beneficial to predict clinical outcomes in patients treated with anti-programmed cell death protein 1 (PD-1) or anti-programmed death-ligand 1 (PD-L1) immunotherapy.^[57]^ A small retrospective study explored the efficacy of radiomics features from ADC to monitor treatment response of bone metastases from PCa, and the findings indicated that the strategy might be utilized as a complement to average ADC values for treatment monitoring.^[58]^

The main characteristics of the studies discussed in Sections 2.4 are detailed in Table 3.

**Table 3:** Performances of radiomics models in treatment response in bone and soft tissue tumors.

Authors	Yr	Study design	Type of tumor	Technique	No. of patients (training + testing)	Performances
Lin et al^[46]^	2020	Retrospective	Osteosarcoma	CT	191	AUC = 0.871, 0.843
Jeong et al^[47]^	2019	Retrospective	Osteosarcoma	PET/CT	70	AUC = 0.82
Huang et al^[48]^	2020	Prospective	Osteosarcoma	MR	12	AUC = 0.97 AUC = 0.90 AUC = 0.81
Kim et al^[51]^	2021	Retrospective	Osteosarcoma	PET/CT	52	The highest test accuracy: 0.85 AUC: 0.89
Crombé et al^[53]^	2019	Retrospective	STS	MR	65	AUC = 0.86
Gao et al^[54]^	2020	Prospective	STS	MR	30	AUC = 0.85
Blackledge et al^[55]^	2019	Prospective	STS	MR	18	Accuracy = 98.1%
Tian et al^[56]^	2014	Prospective	STS	CT	20	Sensitivity: 84.6% Specificity: 71.4%
Sun et al^[57]^	2018	Retrospective	Advanced solid tumors	CT	135	AUC = 0.74 AUC = 0.67
Reischauer et al^[58]^	2018	Prospective	Prostate cancer bone metastases	MR	17	

STS = soft tissue sarcoma.

### 2.5. Prognostic prediction of bone and soft tissue tumors

The prognostic prediction of bone and soft tissue tumors is of vital importance. As a method to reflect tumor heterogeneity, radiomics offers new ideas for tracking recurrence and metastasis of bone and soft tissue tumors in clinical practice. Such capability may be beneficial to treatment selection, patient stratification and justify a therapy switch, which is the reason why clinicians take an interest in survival prediction.^[32]^

The role of radiomics in predicting prognosis was reviewed and the possibility and the significance of integrating these methods into clinical care of patients were discussed. For high-grade osteosarcoma (HOS), the gold treatment standard is NAC, followed by surgical resection and adjuvant chemotherapy.^[59]^ Patients with localized osteosarcoma have significantly improved their long-term survival rates after the introduction of NAC, with 5-year survival rates reaching 60% to 70%.^[60]^ However, a proportion of those patients who respond poorly to NAC still have a poor prognosis. Thus, the accurate prediction of histologic responses to NAC in patients with HOS is the key to selecting treatment plan and evaluating prognosis.^[61]^ A retrospective study validated the hypothesis that a clinical radiomics model exhibited a greater predictive performance compared with clinical factors alone in terms of prognosis, contributing to making individual survival predictions and arranging reasonable follow-up intervals, thereby preventing unnecessary medical resources and expenses.^[62]^ Another study proposed a radiomics nomogram containing the radiomics signature and clinical risk factors for patients with osteosarcoma. Totally, 103 radiomics features were extracted from DWI-MR images and reduced to 8 features by LASSO regression to accurately predict OS, with C-index amounting to 0.813. When evaluating localized osteosarcoma, the union model (clinical + radiomics) could increase the value of the evaluation over clinical factors alone.^[63]^ In terms of predicting early recurrence of osteosarcoma, Liu et al^[64]^ discovered that hemoglobin (HGB) level and joint invasion are effective for predicting relapse risk in osteosarcoma patients; hence, HGB level and joint invasion were used to develop the clinical nomogram, and the radiomics nomogram was developed by fusing the radiomics signature and clinical-based risk factors. The ability to predict the recurrence of osteosarcoma could be improved by a radiomics nomogram, as this multicenter study shows.

Prognostic judgment is an important factor in selecting treatment options for patients with STS, including the need for extended resection and postoperative radiotherapy.^[31]^ In recent years, a series of studies have concentrated on radiomics to determine the prognosis of STS. One study fused PET and MR images of patients with STS to predict clinical outcomes. Image-level fusion outperformed other fusion techniques in terms of prediction performance.^[65]^ Radiomics was explored by Crombé et al,^[66]^ who examined the method in predicting prognosis of patients with locally advanced STS, and the best outcomes could be achieved when relative changes in radiomics features (rRFs) rather than parametric radiomics features (pRFs) were included in DCE-MRI-based models. A quantitative strategy was also proposed to enhance the prediction of patients’ prognosis by these scholars,^[67]^ who developed a radiomics model to predict metastatic relapse in patients with myxoid/round cell liposarcomas (MRC-LPS). It is noteworthy that the best performance was achieved by adding radiomics analysis on a pertinent MRI sequence. There is an emerging body of evidence that MRI-based radiomics approaches have the potential to improve the prediction of survival of STS patients. A hybrid model based on both clinical and radiomics features exhibited an optimal performance, which denotes a promising and easily accessible biomarker for patients with STS.^[68,69]^ According to another research, the radiomic feature Imc1 serves as a predictor of metastatic dissemination in patients with soft tissue sarcomas.^[70]^ A prospective study of 11 patients examined the surveillance efficacy of MRI-based radiomics models concentrating on local recurrence of STS. Three models based on distinct MRI sequences had AUC values ranging from 0.71 to 0.96. T1W had an inferior surveillance performance than T2WI with fat-saturation and T1WI post-Gadolinium administration.^[71]^

The chordoma is prone to local recurrence and has a poor prognosis, with a low progression-free survival (PFS) rate of 5-year progression-free survival.^[72,73]^ Several variables, such as complete surgical resection,^[74]^ post-operative radiotherapy,^[75]^ postoperative proton-beam therapy^[76]^, PBRM1,^[77]^ have been linked to prognosis. With the use of these prognostic factors, a few prediction models were created. Wei et al^[78]^ evaluated the predictive performance of MRI-based radiomics signature as a prognostic biomarker for SBC with a high 95%CI. Similar to this study, another one has demonstrated that radiomic analysis can successfully assist neurosurgeons in modifying the evaluation of patients with a clival chordoma.^[79]^ In terms of predicting early recurrence of bone tumors, according to the low incidence of giant cell tumor of bone (GCTB) in the spine, a few relevant studies were conducted, making it difficult to predict the recurrence for clinicians. Wang et al^[80]^ used SVM to select features, then developed the radiomics model using pre-operative CT images and assessed its performance using 10-fold cross-validation. The research indicated the need for radiomics in order to identify the likelihood of postoperative recurrence. In another study, analysis of texture and shape in several sequences (T1WI, T2WI, DWI, CE-T1WI) at 3.0 Tesla satisfied demand for the estimation of early recurrence of pelvic chondrosarcoma (CS). Five distinct models were developed. The best outcomes were obtained by a clinical radiomics model based on integrating imaging features (T1W + T2W + CET1W) and clinical data.^[81]^

The main characteristics of the studies discussed in Sections 2.5 are detailed in Table 4.

**Table 4:** Performances of radiomics models in prognostic prediction of bone and soft tissue tumors and prediction of bone metastasis.

Authors	Yr	Study design	Type of tumor	Technique	No. of patients (training + testing)	Performances
Wu et al^[62]^	2018	Retrospective	HOS	CT	150	AUC = 0.86, 0.84
Zhao et al^[63]^	2019	Retrospective	HOS	MR	112	C-index = 0.813
Liu et al^[64]^	2021	Retrospective	Osteosarcoma	CT	80	C-index = 0.779, 0.710
Zhao et al^[65]^	2022	Retrospective	STS	PET/MR	51	AUC = 0.9524
Crombé et al^[66]^	2020	Retrospective	STS	MR	50	AUC = 0.87
Crombé et al^[67]^	2020	Retrospective	Myxoid/round cell liposarcoma	MR	35	AUC = 0.64–0.94
Chen et al^[68]^	2021	Retrospective	STS	MR	62	AUC = 0.791 C-index = 0.781
Spraker et al^[69]^	2019	Retrospective	STS	MR	226	C-index = 0.78
Giraudo et al^[70]^	2022	Retrospective	STS	MR	36	Accuracy = 76.7%
Tagliafico et al^[71]^	2019	Prospective	STS	MR	11	AUC = 0.96
Wei et al^[78]^	2019	Retrospective	Chordoma	MR	148	Accuracy = 82.4%
Zhai et al^[79]^	2022	Retrospective	Chordoma	MR	174	AUC = 0.747, 0.807, 0.904 AUC = 0.582, 0.852, 0.914
Wang et al^[80]^	2021	Retrospective	Giant cell tumor of bone	CT	62	AUC = 0.78 Accuracy = 89%
Yin et al^[81]^	2019	Retrospective	Chondrosarcoma	MR	103	AUC = 0.891 ACC = 0.857
Hinzpeter et al^[82]^	2022	Retrospective	Prostate cancer bone metastases	PET/CT	67	Accuracy = 90% Sensitivity = 91% Specificity = 88%
Wang et al^[83]^	2019	Retrospective	Prostate cancer bone metastases	MR	176	AUC = 0.898
Zhang et al^[84]^	2020	Retrospective	Prostate cancer bone metastases	MR	116	AUC = 0.93, 0.92
Chen et al^[85]^	2022	Retrospective	NSCLC bone metastases	CT	195	AUC = 0.82, 0.73
Filograna et al^[86]^	2018	Retrospective	Vertebral bone marrow metastatic	MR	8	T1: AUC = 0.8141 T2: AUC = 0.9116

HOS = high-grade osteosarcoma, STS = soft tissue sarcoma.

### 2.6. Prediction of bone metastasis

Radiomics analysis has also been used to predict the incidence of bone metastasis in osteolytic malignancies, such as PCa. PCa is a major health problem in developed countries owing to its rising prevalence among the elderly.^[87]^ The bones are the most prevalent distant metastatic location for PCa.^[88]^ Bone metastasis, a major cause of pain and mortality in PCa patients, can affect around 30% of PCa patients approximately,^[89]^ and beyond that, the extent of bone metastasis is critical in determining the optimal treatment strategy.^[90]^ It has also been demonstrated that the degree of bone metastatic deserves an independent prognostic factor for PCa.^[91]^ Hinzpeter et al^[82]^ performed volumetric segmentation manually and developed a CT-based radiomics model for differentiating invisible bone metastasis with naked eyes using CT images from unaffected bone using PET images. It was revealed that radiomics has noticeably attracted researchers’ attention in terms of differentiation between bone metastasis and bone metastasis-free.

Emerging evidence has indicated that using CAD for the quantification of risk in bone metastasis via radiomics-based textural analysis is promising. There exists a study looking at preoperative personalized BM prediction in patients with PCa. The predictive biomarker was validated using multivariate logistic regression analysis. The radiomics model, incorporating both combined MR textural features of the primary tumor and clinical risk factors, was validated as a novel approach to facilitate individualized prediction of BM,^[83]^ which is identical to Zhang et al‘s findings.^[84]^

In another study, radiomics was evaluated as a prediction tool, and the top 7 CT-based radiomics features were finally retained through LASSO regression model. The study concluded that the combination of CT radiomics data and clinical imaging features provided an excellent prediction performance for the occurrence of bone metastasis in patients with non-small cell lung cancer (NSCLC).^[85]^ Radiomics features derived from spinal bone marrow have been found to be associated with the risk of the cancer progression. Filograna et al^[86]^ investigated the hypothesis that quantitative imaging features from preradiotherapy MR images (T1WI and T2WI) could predict the incidence of spinal bone marrow metastasis in cancer patients untreated with radiotherapy. Predictors for T1W and T2W images exhibited the best performance, with 2 features for T1W images and 2 features for T2W images.

The main characteristics of the studies discussed in Sections 2.6 are detailed in Table 4.

### 2.7. Application of radiomics in molecular diagnosis

Changes of tumors in genes and proteins can be reflected in macroscopic medical images.^[92]^ Values in molecular diagnosis has received considerable critical attention by a growing number of academics recently. The ultimate objective of these studies is to develop specific imaging biomarkers based on the combination of gene phenotype and phenotypic image analysis. Meyer et al^[93]^ studied the correlation between radiomics features and tumor proliferation index Ki-67, and pointed out that 2 features, 45dgr_RLNonUni in plain T1WI and S(4,0) SumAverg in T2WI, could be used as novel biomarkers to indirectly predict the proliferative capacity of STS. Subsequently, a study used radiomics features derived from MRI to predict MDM2 gene amplification, thus distinguishing liposarcoma (WDLPS) from lipoma. The mean AUC for the radiomics model using only T1W imaging features was 0.83. The model was enhanced to a mean AUC of 0.89 when the T2W imaging features were included. Both of them outperformed the scores of 3 experienced radiologists. The radiomics is crucial to detect lesions at an early stage so that patients can receive an appropriate treatment in a timely manner.^[94]^ Emerging evidence indicated that macroscopic heterogeneity assessed by medical imaging can reflect the aggressiveness of the biological phenotype. In patients with GCTB, Wang et al^[95]^ evaluated the effects of VEGF and P53 on the prediction of PFS in GCTB patients. The preoperative CT imaging was also taken to establish models for predicting these 2 biomarkers. In patients with primary lung adenocarcinoma, Shen et al^[96]^ derived CT imaging-based histogram features of bone metastases with EGFR mutation. It was proposed that the CT histogram features represented imaging markers of EGFR. The presence of the EGFR mutation was substantially correlated with 3 histogram features, including range, skewness, and quantile 0.975. After combining range and skewness, the highest AUC value was achieved (AUC 0.783), as well as a high sensitivity (0.708) and specificity (0.788).

The main characteristics of the studies discussed in Sections 2.7 are detailed in Table 5.

**Table 5:** Performances of radiomics models in application of radiomics in molecular diagnosis.

Authors	Yr	Study design	Type of tumor	Technique	No. of patients (training + testing)	Performances
Meyer et al^[1]^	2019	Retrospective	STS	MR	29	AUC = 0.90
Vos et al^[2]^	2019	Retrospective	Well differentiated liposarcomas & Lipomas	MR	116	AUC = 0.89
Wang et al^[3]^	2022	Retrospective	Giant cell tumor of bone	CT	80	AUC = 0.88, 0.77
Shen et al^[4]^	2019	Retrospective	Primary lung Adenocarcinoma bone metastases	CT	57	AUC = 0.783 Sensitivity = 0.708 Specificity = 0.788

STS = soft tissue sarcoma.

1.Meyer HJ, Renatus K, Hohn AK, et al. Texture analysis parameters derived from T1-and T2-weighted magnetic resonance images can reflect Ki67 index in soft tissue sarcoma. *Surg Oncol*. 2019;30:92–97.

2.Vos M, Starmans MPA, Timbergen MJM, et al. Radiomics approach to distinguish between well differentiated liposarcomas and lipomas on MRI. *Br J Surg*. 2019;106(13):1800–1809.

3.Wang Q, Zhang Y, Zhang E, et al. A multiparametric method based on clinical and CT-based radiomics to predict the expression of p53 and VEGF in patients with spinal giant cell tumor of bone. *Front Oncol*. 2022;12.

4.Shen T, Liu L, Li W et al. CT imaging-based histogram features for prediction of EGFR mutation status of bone metastases in patients with primary lung adenocarcinoma. *Cancer Imaging*. 2019;19(1):34.

## 3. Current status and limitations of radiomics

Radiomics is an inherently product of multidisciplinary intersection of computer vision, machine learning and medical imaging, which combines quantitative and visual evaluation, while saving radiologists’ time.^[97]^ The applications of radiomics in the musculoskeletal system represent an emerging and growing field,^[98]^ which is mainly reflected in tumor differentiation, classification and prediction of treatment response and prognosis, providing an essential tool for precision therapy.^[99]^

Radiomics has some shortcomings that are summarized in the following. A lack of repeatability. There are 5 processes that make up the radiomics workflow. Differences in any of these particular processes might lead to variations in findings.^[2]^ Images are typically acquired from various sequences, and they are reconstructed using different software. In a systematic review, Larue et al discussed effects of various image acquisition parameters on repeatability and quality of features retrieved from different modes, such as CT, PET and MRI.^[97]^ Regarding the existence of numerous morphological changes in tumors, these changes are mainly irregular, and the partial volume effect may obscure the margin of tumors, making it difficult to be well defined in medical images. It is easy to be subjective if images are segmented manually. Furthermore, different dimensionality reduction and statistical methods may lead to different results because data mining after feature extraction requires dimensionality reduction and statistical knowledge of modeling. Inadequate mutual verification. In spite of the fact that the results of radiomics studies in variety of diseases are encouraging, the number of samples is still small, hindering verification of results in various studies. Radiomics studies require a large-scale database, however, in the meantime, radiomics studies on bone and soft tissue tumors are mainly small-scale studies. Lack of samples may make model prediction less accurate and increase the likelihood of overfitting.^[100]^ Unfulfilled automatic and standardized processing. The processes of image segmentation and data processing have not yet been fully automated and standardized, thus, it is essential to make use of advanced deep learning and artificial intelligence methods to achieve fully automated analysis, and to simultaneously establish a standardized quality control and evaluation system to make it mature and eventually applied in clinical settings.^[2,101]^ Radiomics requires substantial imaging datasets. Establishing an imaging database should guarantee patients’ privacy, such as erasing or swapping out patients’ identity information in the original Digital Imaging and Communications in Medicine (DICOM) files. Exploration of relationships between various forms of data is another concern as it enables quick retrieval of all types of data by connecting imaging data with pathology, genetic data, etc.

## 4. Conclusions

Bone and soft tissue malignancies have historically been a diagnostic challenge, and numerous scholars have long attempted to find highly reliable solutions, and the rise of radiomics is a great benefit to them. Radiomics is an emerging and extremely inventive field of research aiming to extract mineable high-dimensional data from clinical images. This review aimed to assist oncologists and radiologists in comprehending and popularizing radiomics, collaborating with scientists to conduct more in-depth studies, as well as highlighting its benefits for cancer patients. Radiomics has great application potential in bone and soft tissue tumors, but a lack of standardization of feature extraction hinders standardization and clinical translation. The Imaging Bio-markers Standardisation Initiative (IBSI) should be commended for the efforts in relation to the problem.^[102]^ The goal of the endeavor is to standardize definitions of usual radiomic features and their calculation. The study only reached a consensus on 172 features in order to keep the scope manageable, and further research is required to hone them. The IBSI is presently working to overcome the restriction. In conclusion, the IBSI was successful in generating and validating reference values for radiomics features. These reference values enable radiomics software verification, facilitating clinical translation of radiomics. As a result, we strongly advise evaluating the IBSI compliance of libraries and software before employing them in research. Additionally, it is necessary to eliminate the limitation of identifying correlation rather than causality, which requires the development of more prospective studies and animal experiments.^[3]^ Furthermore, the importance of radiomics in this field has been confirmed by a great number of studies, while it is still vital to figure out how to effectively apply radiomics to diagnostic imaging and clinical settings. Over the past decade, its system has become increasingly sophisticated, and it is anticipated to combine genomics, metabonomics, transcriptomics, proteomics, immunohistochemistry, etc in the next decade, so that it can develop to a real auxiliary diagnostic tool and eventually realize precision medicine. It is widely accepted that, with the efforts of scholars at home and abroad, radiomics can be integrated with new technical methods, and radiomics can continue to mature and revitalize the development of medical imaging.

## Author contributions

**Methodology:** Jie Peng.

**Project administration:** Xiaohan Zhang, Guanghai Ji.

**Software:** Jie Peng, Hao Xiong.

**Supervision:** Tian Li.

**Validation:** Xiaohan Zhang, Bo Li.

**Writing – original draft:** Guanghai Ji, Tian Li, Bo Li, Hao Xiong.
